# Reintroduction of 5-Fluorouracil Post-cardiac Arrest Secondary to Chemotherapy-Induced Cardiotoxicity

**DOI:** 10.7759/cureus.34232

**Published:** 2023-01-26

**Authors:** Kathie Wu, Prianka Bhattacharya

**Affiliations:** 1 Internal Medicine, Geisinger Medical Center, Danville, USA; 2 Hematology/Oncology, Geisinger Medical Center, Danville, USA

**Keywords:** chemotherapy-induced cardiotoxicity, cardiac arrest, 5-fluorouracil, toxicity, chemotherapy, rectal cancer, out-of-hospital cardiac arrest

## Abstract

5-fluorouracil (5-FU) has been known to have cardiotoxic side effects, including coronary vasospasm, myocardial infarctions, heart failure, arrhythmias, and cardiac arrest. These cases have been reported in patients with either known coronary disease or known risk factors. In cases of acute cardiotoxicity, cessation of fluoropyrimidines is recommended, and reintroduction of the medication is generally avoided. We present a case of a young patient with no known risk factors for coronary disease, who presented with an acute cardiac arrest suspected secondary to vasospasm from the administration of 5-FU for the treatment of rectal cancer and was successfully maintained on treatment with 5-FU post-arrest after transitioning from an infusion to bolus administration.

## Introduction

Fluoropyrimidines, particularly 5-fluorouracil (5-FU), are commonly used for the treatment of glandular and squamous carcinomas, including the head and neck, esophagus, stomach, and bladder, and are standard of care for the treatment of advanced colorectal cancers [1]. Despite the known risks of cardiac toxicity, the incidence has been low, ranging from 1% to 19% [2]. Of these cases, the most common manifestations of cardiotoxicity are angina or acute coronary syndromes. Less common are arrhythmias, myocarditis, and, in very few cases, death [2,3].

## Case presentation

**Figure 1:**
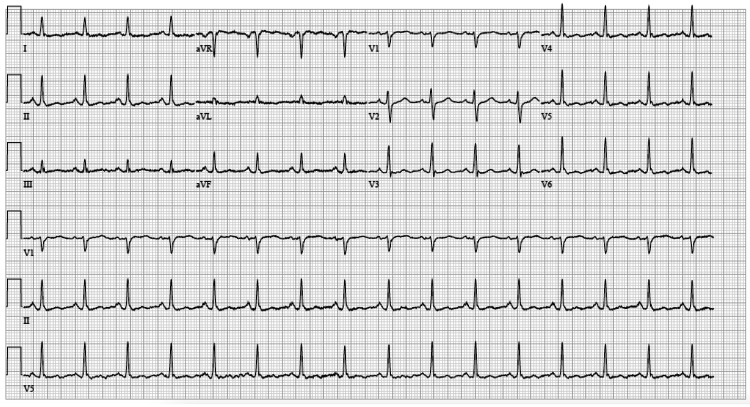
Admission ECG with no notable ST or T wave abnormalities or corrected QT prolongation

**Figure 2:**
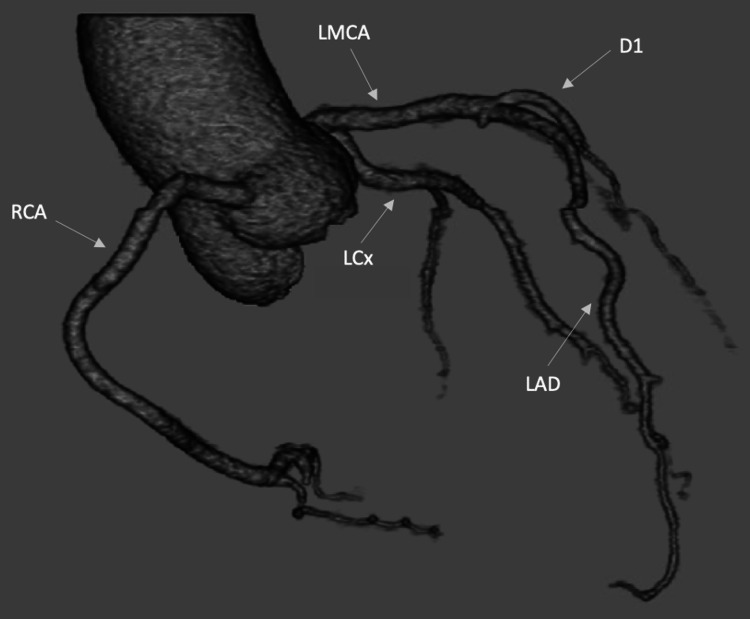
Three-dimensional reconstruction of coronary CT scan with no evidence of coronary disease RCA: right coronary artery; LMCA: left main coronary artery; LCx: left circumflex artery; LAD: left anterior descending artery; D1: diagonal artery.

A 30-year-old female with a past medical history only notable for stage IV rectal adenocarcinoma with metastasis to the liver and bone on infusional fluorouracil, leucovorin, and oxaliplatin (FOLFOX) and bevacizumab presented to the hospital after she was found unresponsive at home. On arrival, she was noted to be in ventricular fibrillation cardiac arrest, which transitioned to pulseless electrical activity (PEA) arrest after an initial shock, and subsequent conversion to sinus tachycardia and achievement of return of spontaneous circulation (ROSC) after a second defibrillation. Her notable history was being disconnected from her 5-FU pump earlier that same day. On admission, targeted temperature management was initiated and she was intubated for airway protection. Her electrocardiogram (ECG) showed no ischemic changes and normal corrected QT (QTc) (Figure 1). Chest CT showed no evidence of acute pulmonary embolism. The transthoracic echocardiogram obtained showed a reduced ejection fraction of 25%. Though suspicion of ischemia was low, the patient underwent a coronary CT with no evidence of coronary artery disease (Figure 2). At this time, suspicion for fluoropyrimidine including coronary vasospasm leading to cardiac arrest was the leading differential. Per discussion with oncology, the decision was made to transition the patient from FOLFOX to bolus 5-FU, leucovorin, and oxaliplatin (FLOX) regimen to eliminate the infusion component of 5-FU and maintain the bolus. The decision was made to discontinue bevacizumab given the increased risk for vascular toxicity, particularly with her presentation of coronary vasospasm and arrest. Consideration was given to transitioning her therapy to CAPOX (capecitabine plus oxaliplatin), but the patient had progressed to metastatic disease to the liver while on capecitabine a few months prior. Her chemotherapy cycle was arranged to be given inpatient with close telemetry monitoring after the 5-FU bolus gave her a cardiotoxic event. The patient was started on nitrates and calcium channel blockers for pretreatment prior to chemotherapy administration. Her ECG, telemetry, and cardiac enzymes remained unremarkable after her first cycle of the modified regimen with 5-FU bolus. Her repeat echocardiogram after the arrest showed improvement in ejection fraction back to 60%. However, given the concerns that the patient may develop recurrent adverse events with the continued use of fluoropyrimidine despite the administration changes, and that pre- and post-treatment ECGs would not be sufficient in predicting another event, the decision was made to have the patient admitted for monitoring with each cycle of FLOX therapy. To date, the patient has been tolerating treatment and no further adverse events have occurred.

## Discussion

Fluoropyrimidines have many known common side effects, including diarrhea, anorexia, and mucositis. Though less common, cardiotoxicity can also occur, with effects ranging from nonspecific ECG changes to cardiomyopathy and even cardiac death [4,5]. This risk increases in patients who are undergoing concurrent radiation therapy, multi-agent chemotherapy, and those with underlying coronary artery disease or structural heart disease [6]. Interestingly, traditionally known risk factors for ischemic heart disease such as hyperlipidemia, tobacco use, and diabetes were not shown to have an association with cardiotoxicity [7]. The mode of administration of chemotherapy, whether bolus versus infusion, has also been shown to have varying risks of cardiotoxicity, with most cases of toxicity occurring in continuous 5-FU infusions rather than bolus [4]. Though the exact mechanism of cardiotoxicity remains unclear, theories include coronary artery vasospasm, toxicity to the myocardium, endothelial dysfunction, and hypercoagulability [6]. In the case of coronary vasospasm, patients may present with symptoms of acute coronary syndrome including ECG with ST changes or a rise in troponin levels, but coronary angiography usually reveals no evidence of disease. One study showed that increasing doses of 5-FU may induce protein kinase C-mediated endothelium vasoconstriction, leading to vasospasm [8]. Another study showed high levels of endothelin-1, a vasoconstrictor in coronary artery disease, in patients with 5-FU-induced cardiotoxicity [9]. There have been several pharmacological interventions including non-dihydropyridine calcium channel blockers and nitrates that have been used both in the prevention and for the treatment of symptoms associated with vasospasm with variable success [10,11]. Transitioning from a 5-FU infusion to a bolus has also had limited evidence [4,11]. In addition, the risk of recurrence of cardiotoxicity when patients are reintroduced to 5-FU therapy after an initial insult is as high as 82% to 100% [12]. Therefore, re-challenging with fluoropyrimidines after an initial event should only occur after careful consideration of alternative regimens and weighing the chances of survival with the risk of recurrent cardiac event, particularly when the presentation was cardiac arrest. Our patient had no predilection for cardiotoxicity given her lack of history of coronary or structural heart disease. Her presentation of cardiac arrest was also at the severe end of the spectrum in terms of adverse events, which made the decision for the reintroduction of 5-FU particularly challenging. However, given her advanced disease, the decision was made to modify her treatment plan to eliminate the infusion portion of the chemotherapy regimen rather than complete termination of therapy with 5-FU. She was pretreated with both calcium channel blockers and nitrates to mitigate any effects of coronary vasospasm and monitored inpatient with every cycle given her history of arrest. To date, our patient has had appropriate tolerance with these modifications despite the scarcity of literature on success with the reintroduction of therapy with 5-FU, particularly with this severity of cardiotoxicity.

## Conclusions

Fluoropyrimidine-induced cardiotoxicity can have varying degrees of severity, but vigilance must be maintained in the monitoring of any relevant symptoms, and we must have a high index of suspicion even in patients with no known history of heart disease. Though early cessation and supportive therapy have been the mainstay of treatment in literature, the reintroduction of fluoropyrimidines in patients even with a history of cardiotoxicity may be considered with adaptations in therapy and preventative measures. However, this decision should be made after careful consideration of alternative therapies and with shared discussions with the patient.
